# Normal-weight obesity in high-altitude youth: Gender disparities and protective effects of native adaptation

**DOI:** 10.1371/journal.pone.0328992

**Published:** 2025-08-01

**Authors:** Nanhui Peng, Jia Li, Baifang Liu, Xu Yan, Nima Ouzhu

**Affiliations:** 1 Exercise and Virtual Simulation Laboratories, College of Physical Education, Southwest University, Chongqing, China; 2 Institute for Health and Sport, Victoria University, Melbourne, Australia; 3 Tibet Police Academy, Lhasa, Tibet, China; 4 Australian Institute for Musculoskeletal Science (AIMSS), Melbourne, Australia; 5 Department of Medicine-Western Health, The University of Melbourne, Melbourne, Australia; 6 College of Education, Tibet University, Lhasa, Tibet, China; Endocrinology and Metabolism Population Sciences Institute, Tehran University of Medical Sciences, IRAN, ISLAMIC REPUBLIC OF

## Abstract

**Background:**

While high altitude has been associated with reduced muscle mass, its effects on fat mass remain controversial, with studies reporting both fat accumulation and loss. Normal weight obesity (NWO), characterized by normal body mass index (BMI) but excessive body fat, is an emerging metabolic health concern. This study aimed to investigate the prevalence of NWO among young adults in a high-altitude region, while also analyzing body composition differences between genders, as well as native versus migrant populations.

**Methods:**

In this cross-sectional study, 1,313 university students (mean age: 19.6 ± 1.6 years; 719 females and 594 males; all enrolled in undergraduate studies) from Lhasa, Tibet (altitude: 3,650m), who voluntarily participated in body composition measurements during the university’s annual physical fitness assessments. The participants were from multiple academic departments. They underwent anthropometric and body composition assessments, including body fat percentage (BF%), fat-free mass (FFM), skeletal muscle mass (SMM), and waist-to-hip ratio (WHR). Participants were stratified by BMI and BF% to determine NWO prevalence, with subgroup analyses for gender and residential background.

**Results:**

BF% trends varied by gender; female students showed a linear increase with BMI, while male students exhibited a phased pattern with two turning points at BMI = 19.6 and 26.5. The overall prevalence of NWO among high-altitude university students was 22.2%, accounting for 57% of individuals with excessive body fat. Females were disproportionately affected (27.7% vs.16.7% in males). Both females and males with NWO had significantly lower FFM and SMM than their non-NWO counterparts (P < 0.001). A subset analysis of 389 individuals revealed that NWO incidence was significantly lower among high-altitude native females and males compared with their migrant counterparts. Among those with NWO, migrant females had a higher BF% (P = 0.02), whereas native males exhibited a higher WHR (P = 0.009).

**Conclusions:**

NWO prevalence among young adults in a high-altitude region was comparable to lowland populations, with notable gender disparities. NWO was associated with reduced muscle mass, suggesting elevated metabolic health risks. The lower NWO prevalence among native high-altitude residents suggests potential protective effects associated with chronic hypoxia adaptation. These findings underscore the need for further research to elucidate the complex relationship between chronic hypoxia, body composition, and metabolic health in high-altitude populations. Such insights are crucial for developing targeted interventions addressing normal weight obesity.

## Introduction

Obesity has emerged as a significant global public health challenge, it is projected that approximately 1 billion people worldwide will be affected by obesity by 2030, including one in five women and one in seven men [1]. Similarly, obesity among youth is a growing concern, with 2022 data from the World Health Organization (WHO) revealing that over 160 million children and adolescents aged 5–19 were affected [2]. Obesity is defined as an abnormal or excessive accumulation of body fat that may impair health [3]. Body Mass Index (BMI), calculated as weight (kg) by height squared (m^2^), is the most widely used metric for defining obesity [4]. According to WHO, a BMI ≥ 25 kg/m² is considered overweight, and a BMI ≥ 30 kg/m² is classified as obese [2]. In addition to BMI, other common measures used to assess obesity include body fat percentage (BF%) and waist circumference (WC). For instance, a BF% > 25% in males and > 32% in females is often used as a threshold for defining obesity. Abdominal obesity, as defined by the National Institutes of Health (NIH), is indicated by a WC > 102 cm in men and > 88 cm in women [2].

Studies have shown that a BMI of ≥ 30 kg/m^2^ demonstrates high specificity for diagnosing obesity, its sensitivity drops to below 50% in individuals with a BMI between 18.5 and 29.9 kg/m^2^. Compared to body fat percentage (BF%) or waist circumference (WC) criteria, BMI produces fewer false positives but substantially more false negatives [5]. This suggests BMI alone may not accurately identify all cases of obesity, especially in people with normal-weight obesity (NWO). NWO refers to individuals who have a normal BMI (18.5 ≤ BMI < 24) but exhibit excessive body fat (> 20% in males and > 30% in females) and low skeletal muscle mass [6]. Research indicates that individuals with NWO have a metabolic syndrome risk comparable to that of obese individuals, with an incidence rate four times higher than those with normal body fat levels [7]. However, because of their normal BMI, up to 83% of these individuals are not diagnosed or treated [8].

Previous research suggests that residents of high-altitude regions tend to have lower rates of overweight and obesity compared to populations at sea level, with prevalence declining as altitude increases [9–11]. However, evidence on how hypoxic exposure affects body composition remain inconsistent. Short-term hypoxic exposure can trigger acute responses, such as increased ventilation, sympathetic nervous system activation, and metabolic alterations, leading to transient effects on glucose metabolism and fat oxidation [12]. In contrast, long-term hypoxic exposure – lasting several weeks, months, or even a lifetime – may produce different effects. Research on Himalayan Tibetans shows lower body fat and abdominal fat distribution at higher altitude [13]. Chronic hypoxia at high altitudes also significantly impacts body composition, often leading to reductions in body weight, fat-free mass (FFM), and skeletal muscle mass (SMM) [14].

However, most existing research on obesity at high altitudes focuses on short-term exposure, such as in hikers and climbers, rather than long-term residents. Moreover, few studies explored variations in body composition responses by gender or differentiate between native and migrant high-altitude populations. By integrating detailed body composition analysis, this study aims to assess obesity classification among young adults living at high-altitude, offering a scientific basis for early identification and targeted intervention strategies.

## Materials and methods

### Study design

This cross-sectional study was conducted from September 26 to October 6, 2024, at Tibet University in Lhasa (altitude: 3,650 meters). The study was approved by the Medical Ethics Committee of Tibet University (Ethics Approval Number: ZDYXLL2024020) and conducted in accordance with the ethical principles outlined in the Declaration of Helsinki. All participants were informed about the study, participated voluntarily, and had their rights and privacy protected throughout the research process. The study aimed to assess the prevalence of normal weight obesity (NWO) among young adults living in high-altitude regions and to examine differences in body composition withing the NWO group based on gender and residential background (Native vs. migrant populations).

### Participants and sampling method

A total of 1,313 university students (719 females and 594 males; mean age = 19.6 ± 1.6 years) participated in this study. Participants were recruited during the university’s annual physical fitness assessment and voluntarily underwent body composition measurements. A quasi-random convenience sampling method was applied, aligned with the university’s testing schedule. Although no a priori sample size calculation was conducted, a post hoc power analysis using G*Power 3.1 indicated that the study had sufficient power (>0.90) to detect medium-sized effect (Cohen’s d = 0.5) at a significance level of α = 0.05.

**Fig 1:**
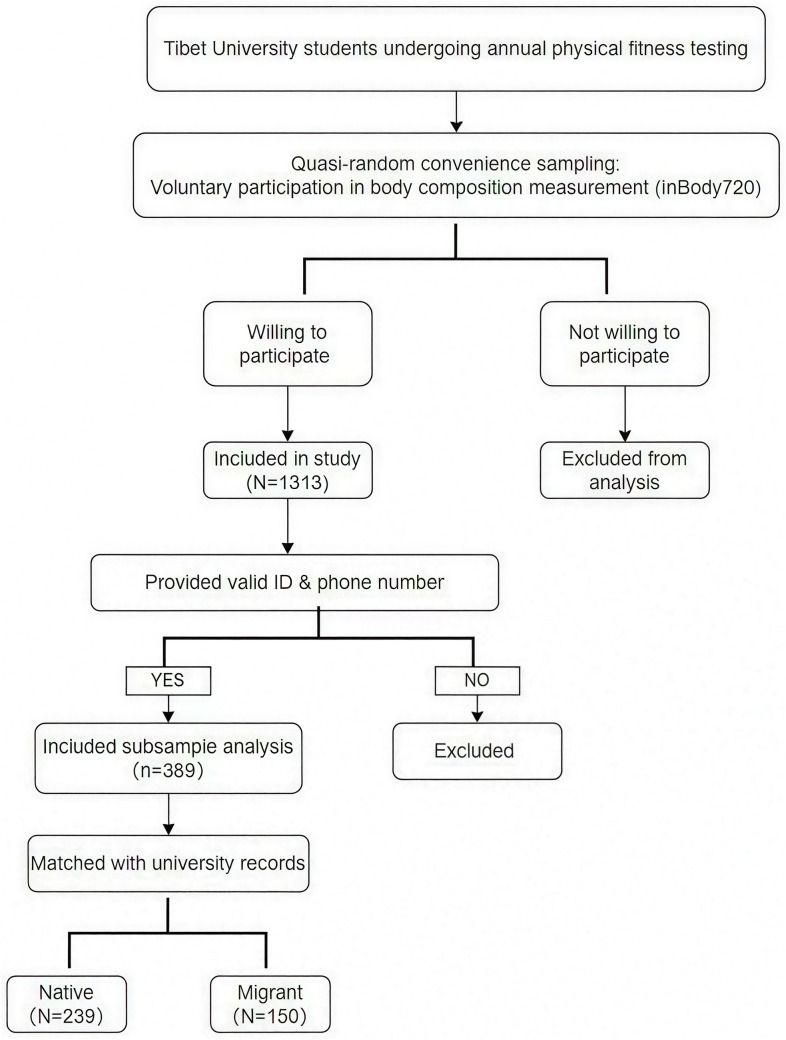
Participant Recruitment and Selection Process.

Of the total participants, 389 voluntarily provided identifiable information (e.g., ID numbers and phone numbers), which was cross-referenced with university administrative records to determine their places of origin. Based on this verification, 389 students were classified by residential background: those with generational residence in high-altitude regions were categorized as natives, while those who had migrated from low-altitude areas to attend university in Lhasa were classified as migrants [15]. Although not all participants provided identifiable information, classification was highly reliable for those who did (Fig 1).

### Definition of variables

BMI categories were defined according to Chinese national guidelines: underweight (<18.5), normal (18.5–23.9), overweight (24–27.9), and obese (≥28). Normal weight obesity (NWO) was defined as having a BMI withing the normal range (BMI < 24 kg/m^2^) accompanied by excessive body fat percentage (BF% > 20% for males, > 30% for females).

### Measurements

Height was measured using a Hengkang Jiaye (HK6800B-ST) instrument with a precision of ±0.2 cm. The device was positioned flat against a wall, with the vertical column properly aligned and securely fastened. During measurement, participants stood barefoot with their heels, lower back, and shoulder blades in contact with the column, maintaining a natural posture. The tester gently lowered the horizontal plate to rest on the top of the participant’s head and recorded the measurement.

Body composition was then assessed using the InBody 720 analyzer, which employs bioelectrical impedance analysis (BIA) to estimate fat-free mass (FFM), skeletal muscle mass (SMM), and waist-to-hip ratio (WHR), providing a comprehensive profiles of body composition.

### Outcome classification

**Fig 2:**
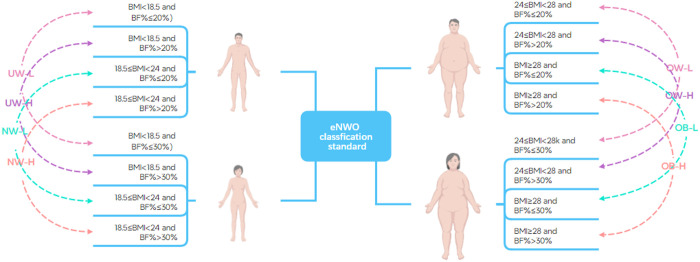
Expanded Normal-Weight Obesity Grouping.

Participants were stratified into eight groups based on the combination of Body Mass Index (BMI) and Body Fat Percentage (BF%), following an expanded classification system for Normal Weight Obesity (eNWO):1) Underweight-Low Fat (UW-L), 2) Underweight-High Fat (UW-H), 3) Normal Weight-Low Fat (NW-L), 4) Normal Weight-High Fat (NW-H), 5) Overweight-Low Fat (OW-L), 6) Overweight-High Fat (OW-H), 7) Obese-Low Fat (OB-L), and 8) Obese-High Fat (OB-H). Individuals with a BMI in the normal or lower range (BMI < 24 kg/m^2^) but an elevated BF% (males > 20%, females > 30%) were classified as having NOW (Fig 2).

### Statistical analysis

Descriptive statistics (mean±SD) were calculated for continuous variables. To examine the relationship between BF% and body mass index (BMI), the study employed a combination of non-parametric visualization and piecewise linear regression techniques.

First, locally weighted scatterplot smoothing (LOWESS) was applied to visualize gender-specific trends in the BMI–BF% relationship. LOWESS is a non-parametric regression method that fits simple models to localized subsets of data, allowing for the detection of nonlinear patterns without assuming a specific functional form [16].

Second, to identify potential inflection points and structural changes in the BMI–BF% relationship among male participants, piecewise linear regression was conducted using the pwlf package in Python. This method fits multiple linear segments to the data and estimates breakpoints between them, based on the least squares criterion [17]. An initial model with three breakpoints was specified, and the two most significant slope changes (i.e., curvature) were selected as final inflection points, allowing for the identification of BMI thresholds at which the rate of BF% increase changed markedly.

Group comparisons across BMI categories, defined by the expanded normal weight obesity (eNWO) classification, were performed using analysis of covariance (ANCOVA), with BMI included as a covariate to adjust for potential confounding in body composition variables such as skeletal muscle mass (SMM). Additionally, independent-sample t-tests were used to assess differences in eNWO-related indicators between residential background groups (i.e., native vs. migrant).

All statistical analyses were performed using SPSS version 27.0, Python, and GraphPad Prism 9.0. A p-value of < 0.05 was considered statistically significant.

## Results

### Participant characteristics

Among the 1,313 participants, 719 were female and 594 were male. The average age was comparable between genders (females: 19.5 ± 1.5 years; males: 19.6 ± 1.8 years). Males had higher average height (173.7 ± 5.9 cm), weight (66.5 ± 13.2 kg), BMI (22.0 ± 3.9 kg/m²), fat-free mass (52.7 ± 6.3 kg), and skeletal muscle mass (28.5 ± 3.8 kg) compared to females. In contrast, females had a higher body fat percentage (28.3 ± 5.7%) than males (19.5 ± 7.8%). WHR was similar between genders. Details are presented in Table 1.

**Table 1:** Summary of demographic and body composition characteristics of male and female participants.

Gender	Sample Size	Age	Height(cm)	Weight(kg)	BMI(kg/m^2^)	BF%	FFM(kg)	SMM(kg)	WHR
Female	719	19.5 ± 1.5	161 ± 5.5	54.3 ± 8.4	21 ± 2.8	28.3 ± 5.7	38.6 ± 4	20.3 ± 2.3	0.82 ± .07
Male	594	19.6 ± 1.8	173.7 ± 5.9	66.5 ± 13.2	22 ± 3.9	19.5 ± 7.8	52.7 ± 6.3	28.5 ± 3.8	0.83 ± 0.7

Note: BMI = Body Mass Index; BF% = Body Fat Percentage; FFM = Fat-Free Mass; SMM = Skeletal Muscle Mass; WHR = Waist-to-Hip Ratio.

### Grouping based on different BMI classification standards

**Fig 3:**
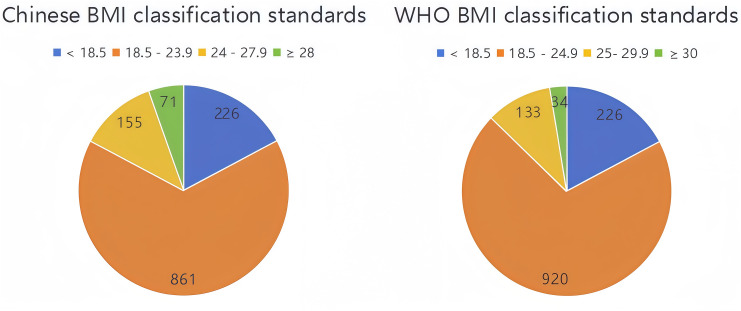
BMI Categories according to Chinese and WHO Standards.

According to the Chinese BMI classification standard, among the 1,313 college students surveyed, 226 were classified as underweight, 861 as normal weight, 155 as overweight, and 71 as obese. These categories were used in all subsequent analyses. For reference and comparison, applying the WHO international BMI classification standard altered the distribution slightly: the number of individuals classified as normal weight increased to 920, while the numbers of those classified as overweight and obese decreased to 133 and 34, respectively (Fig 3).

### Prevalence of normal weight obesity among young adults at high altitude

**Fig 4:**
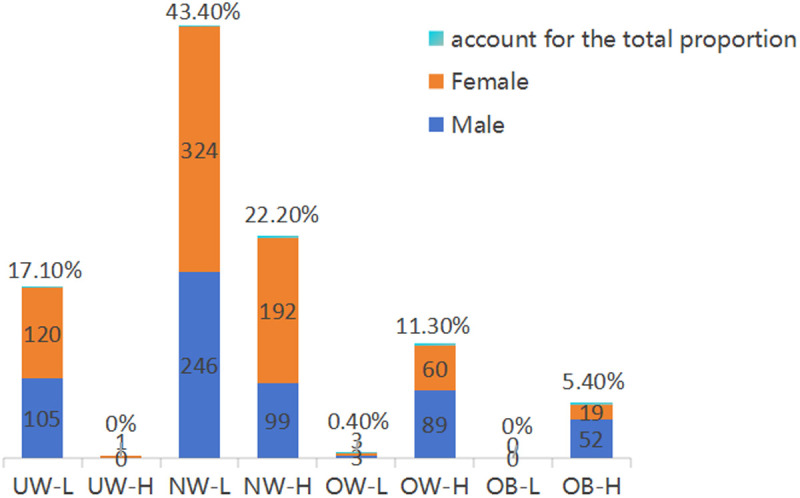
Participant stratification according to the expanded normal weight obesity (eNWO) standards.

A further classification of this population revealed that 225 individuals fell into the UW-L category (female = 120, male = 105), representing 17.1% of the total sample. The UW-H category included only one individual (female = 1). In the NW-L category, 570 individuals (female = 324, male = 246) accounted for 43.4% of the total population, while the NW-H category included 291 individuals (female = 192, male = 99), making up 22.2% of the total. Among overweight individuals, 6 individuals (female = 3, male = 3) were classified as OW-L (0.4%), while 149 individuals (female = 60, male = 89) fell into the OW-H category. The OB-H category comprised 71 individuals (female = 19, male = 52), while no individual was classified in the OB-L category (Fig 4).

Notably, the prevalence of normal weight obesity in our high-altitude college student population was 22.2%, higher than some reported rates in lowland populations. For comparison, a large-scale nationwide survey conducted in China using the data from 2007–2008 reported an NWO prevalence of 7.46% among 23,748 adults aged 20 years or older [18]. Similarly, a study conducted in Kraków, Poland, reported prevalence rates of 16.04% in 2010 and 16.11% in 2020 [19]. In contrast, our reported NWO prevalence was comparable to the 20.1% reported in a 2018 study involving a similar age group, albeit with a smaller sample size [20].

**Fig 5:**
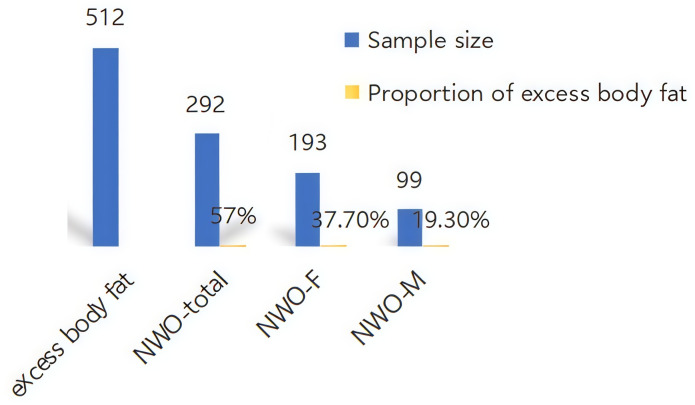
Normal weight obesity in females and males.

Overall, 512 individuals exhibited excess body fat, including 292 individuals classified as having normal-weight obesity (UW-H, NW-H), representing 57% of those. with excess body fat. Among these, 192 females (71.0%) and 99 males (41.3%) are categorized as having normal weight obesity (Fig 5).

In summary, based on China’s BMI standards for obesity screening, the prevalence of overweight and obesity in this population is only 17.0%. However, when incorporating BF% as a criterion, the actual rate of excess body fat rises significantly to 39.0%, with 57% of these individuals classified as having normal weight obesity category. Notably, the incidence of NWO is higher among females than males.

### Gender-specific relationship between BMI and BF%

**Fig 6:**
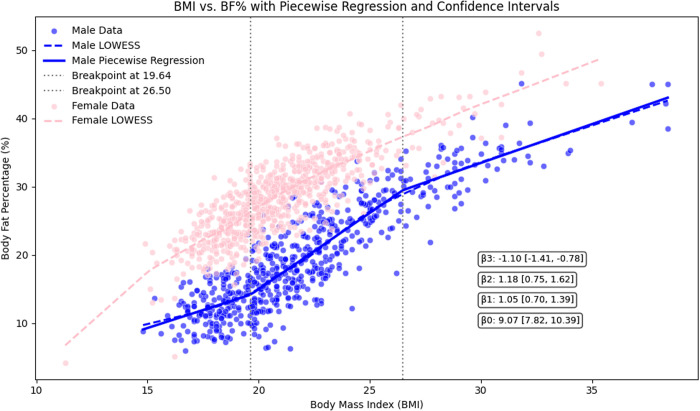
Segmented regression analysis of BMI and BF% in females and males.

Fig 6 presents the distribution of BF% across BMI categories among the 1,313 university students. In females, BF% increased steadily and consistently with rising BMI. In contrast, males displayed a distinct stepwise pattern in the BMI-BF% relationship, with two significant inflection points identified at BMI values of 19.6 and BMI = 26.5 (P < 0.01).

To model this relationship, a piecewise linear regression was applied. For BMI < 19.6, BF% increased at a modest rate (β₁ = 1.05, 95% CI: [0.67, 1.44]). In the mid-range (19.6 ≤ BMI ≤ 26.5), the rate of increase in BF% became more pronounced (β₂ = 1.18, 95% CI: [0.70, 1.66]). However, beyond a BMI of 26.5, the trend reversed, with rate of BF% decreasing as BMI continued to rise (β₃ = −1.10, 95% CI: [−1.43, −0.76]).

### Comparative analysis of body composition among young adults across BMI categories

#### Comparative analysis of body composition among female young adults across BMI categories.

Among normal-weight females, those in the high body fat group (NW-H) exhibited significant differences in body composition compared to the low body fat group. Specifically, BMI and BF% in the NW-H group were 8.4% and 25.8% higher, respectively (P < 0.001), while FFM and SMM were 1.6% and 1.48% lower, respectively (P < 0.001). No significant difference was observed in WHR.

Among overweight females, the OW-H group had a 27.9% higher BF% (P < 0.001), with significantly lower FFM and SMM by 15.5% and 16.5%, respectively (P < 0.001). However, no significant difference in WHR was found between the groups (see Table 2).

**Table 2:** Comparison of body composition in female young adults by comprehensive group classification.

Group	age	Statistical	BMI(kg/m2)	BF%	FFM(kg)	SMM(kg)	WHR
UW-L(n = 120)	19.6 ± 1.54		17.4 ± 1	21.7 ± 3.7	35.7 ± 3.2	18.6 ± 1.7	0.72 ± 0.04
UW-H(n = 1)	\		\	\	\	\	\
NW-L(324)	19.6 ± 1.53		20.2 ± 1.2	26 ± 2.8	38.6 ± 3.3	20.3 ± 1.9	0.81 ± 0.03
NW-H(192)	19.3 ± 1.52		21.9 ± 1.2	32.7 ± 2.1	38 ± 3	20 ± 1.7	0.85 ± 0.03
		P	<0.001	<0.001	<0.001	<0.001	0.106
OW-L(n = 3)	17,7 ± 0.58		24.5 ± 0.4	28 ± 1.9	50.4 ± 6.3	27.2 ± 3.4	0.92 ± 0.003
OW-H(n = 60)	19.5 ± 1.42		25.3 ± 1.1	35.8 ± 3.1	42.6 ± 3.8	22.7 ± 2.2	1 ± 0.003
		P	0.2	<0.001	<0.001	<0.001	0.138
OB-L(n = 0)	\		\	\	\	\	\
OB-H(n = 19)	19.3 ± 1.41		30.3 ± 2.1	42.2 ± 4.5	46.7 ± 5.9	25 ± 3.5	0.82 ± 0.07

Note: UW-L = Underweight–Low Fat; UW-H = Underweight–High Fat; NW-L = Normal Weight–Low Fat; NW-H = Normal Weight–High Fat; OW-L = Overweight–Low Fat; OW-H = Overweight–High Fat; OB-L = Obese–Low Fat; OB-H = Obese–High Fat; BMI = Body Mass Index; BF% = Body Fat Percentage; FFM = Fat-Free Mass; SMM = Skeletal Muscle Mass; WHR = Waist-to-Hip Ratio

### Comparative analysis of body composition among male young adults across BMI categories

Among normal-weight males, those in the high body fat group (NW-H) had FFM and SMM levels that were 1.7% and 1.8% lower, respectively, compared to the low body fat group (NW-L), while their WHR was significantly higher by 6.2% (P < 0.001).

In the overweight group, individuals with high body fat (OW-H) exhibited markedly lower FFM and SMM levels, by 18.4% and 20.4%, respectively, while WHR was 7.1% higher (P < 0.001) (see Table 3).

**Table 3:** Comparison of body composition in male young adults by comprehensive group classification.

Group	age	Statistical	BMI(kg/m^2^)	BF%	FFM(kg)	SMM(kg)	WHR
UW-L(n = 105)	19.1 ± 1.3		17.5 ± 0.7	11.7 ± 2.8	46.6 ± 3.8	24.7 ± 2.1	0.75 ± 0.03
UW-H (n = 0)	\		\	\	\	\	\
NW-L(n = 246)	19.8 ± 1.9		20.6 ± 1.3	15.1 ± 3.2	52.4 ± 4.7	28.3 ± 2.8	0.81 ± 0.04
NW-H(n = 99)	19.9 ± 1.9		22.2 ± 1.2	23.5 ± 2.6	51.5 ± 4.7	27.8 ± 2.7	0.86 ± 0.03
		P	<0.001	<0.001	<0.001	<0.001	<0.001
OW-L(n = 3)	18.3 ± 0.6		25 ± 1.1	15.7 ± 3.2	69.1 ± 4.9	38.7 ± 3	0.85 ± 0.01
OW-H(n = 89)	19.5 ± 1.6		25.6 ± 1	27.6 ± 3.2	56.4 ± 5.4	30.8 ± 3.2	0.91 ± 0.03
		P	0.301	<0.001	<0.001	<0.001	<0.001
OB-L(n = 0)	\		\	\	\	\	\
OB-H(n = 52)	19.7 ± 1.8		30.7 ± 2.8	34.7 ± 4.2	61.5 ± 6.1	33.8 ± 3.6	0.97 ± 0.03

Note: UW-L = Underweight–Low Fat; UW-H = Underweight–High Fat; NW-L = Normal Weight–Low Fat; NW-H = Normal Weight–High Fat; OW-L = Overweight–Low Fat; OW-H = Overweight–High Fat; OB-L = Obese–Low Fat; OB-H = Obese–High Fat; BMI = Body Mass Index; BF% = Body Fat Percentage; FFM = Fat-Free Mass; SMM = Skeletal Muscle Mass; WHR = Waist-to-Hip Ratio

### Comparison of normal weight obesity between native and migrant young adults

**Fig 7:**
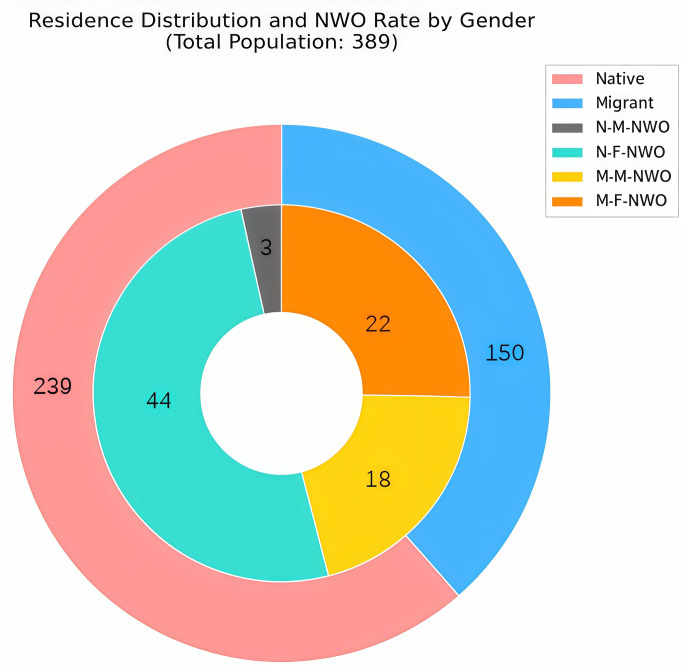
Prevalence of NWO among native and migrant young adults.

To further explore the impact of student origin on NWO, we categorized a subset of 389 college students into two groups: high-altitude natives and migrants. The subset included 239 native students (65 males and 174 females) and 150 migrant students (94 males and 56 females). Further analyses revealed that 47 native students and 40 migrant students were classified as having NWO, with an overall incidence of 19.7% among native students and 26.7% among migrant students (Fig 7). Notably, the prevalence of NWO was significantly lower among both native females (25.3%) and males (4.6%) compared to migrant females (39.3%) and males (19.1%), respectively.

We further categorized this cohort based on BMI, with the results presented in Table 4. Among individuals within the normal BMI range, the prevalence of NWO was 33.6% in native females and 7.5% in native males. In migrants, NWO prevalene was higher, at 52.4% for females and 32.1% for males.

**Table 4:** Stratification by BMI and BF% across residential backgrounds.

Group		Native				Migrants		
	Female (n = 174)	Age	Male (n = 65)	Age	Female (n = 56)	Age	Male (n = 94)	Age
UW-L	30	_20.3 ± 1.7_	16	_19.3 ± 1.1_	9	_19 ± 1.2_	18	_19.1 ± 1.9_
NW-L	87	_19.7 ± 1.4_	37	_19.5 ± 1.3_	20	_19.8 ± 1.1_	38	_19.5 ± 2.0_
NW-H	44	_19.6 ± 1.6_	3	_19.7 ± 0.6_	22	_18.9 ± 1.2_	18	_19.5 ± 2.1_
OW-L	1	\	1	1	0	\	0	1
OW-H	9	_19.9 ± 1.1_	6	_20.2 ± 1.2_	4	_19.3 ± 1.5_	11	_20.6 ± 2.6_
OB-H	3	20.3 ± 0.6	2	20.5 ± 0.7	1	18.0	9	18.8 ± 1.4

Note: UW-L = Underweight–Low Fat; UW-H = Underweight–High Fat; NW-L = Normal Weight–Low Fat; NW-H = Normal Weight–High Fat; OW-L = Overweight–Low Fat; OW-H = Overweight–High Fat; OB-L = Obese–Low Fat; OB-H = Obese–High Fat.

**Fig 8:**
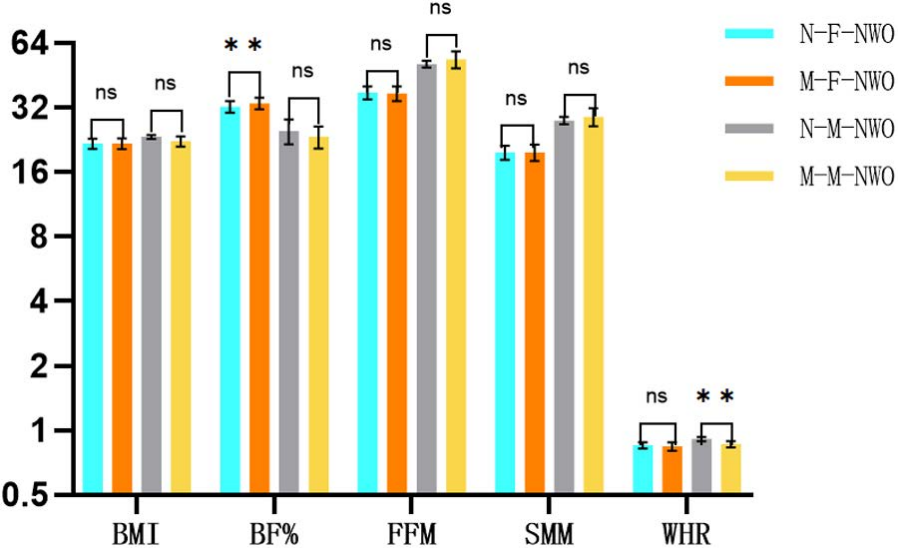
Differences in Body Composition of NWO Across Different Living Arrangements.

There were no significant differences in BMI, FFM, SMM, or WHR between native and migrant females with NWO. However, BF % was significantly higher inmigrant females, exceeding the native females by 3.7% (P = 0.02). For males with NWO, no significant differences were observed in BMI, body fat percentage, FFM, or SMM between native and migrant groups. However, WHR was significantly higher in native males, surpassing that of their migrant counterparts by 5.8% (P = 0.009, Fig 8).

## Discussion

This study assessed the body composition of 1,313 young adults in a high-altitude region to investigate the prevalence of NWO and associated differences in body composition. The findings revealed that 22.2% of this had NWO, accounting for 57% of those with excess body fat, with a significantly higher prevalence in females. This NWO prevalence was comparable to the 20.1% reported in a 2018 study involving a similar age group, albeit with a smaller sample size [20]. These results highlight the limitations of BMI as a sole indicator of obesity-related health risks. The body composition characteristics of the NWO group— marked by high BF%, high WHR, and low SMM—suggest an elevated risk for metabolic syndrome. Although overweight and obese individuals also have an excessive amount of body fat, they often retain more SMM compared to individuals with NWO. In contrast, NWO individuals exhibit a unique risk profile, as they have lower skeletal muscle mass and a higher BF%, which results in a greater likelihood of metabolic dysfunction despite being classified as having a normal BMI. This imbalance in body composition is linked to an increased risk of cardiovascular diseases, type 2 diabetes, and other metabolic disorders [7,21].

This study assessed obesity using BMI thresholds from both the Chinese Ministry of Health and WHO international standards, revealing some discrepancies in classification. Under Chinese criteria, 17.2% of young adults were classified as overweight or obese. However, when applying WHO international BMI standards,this rate dropped to 12.7% (167 out of 1,313). This contrast (17.2% vs. 12.7%) underscores how region-specific BMI thresholds can skew obesity prevalence estimates compared to global benchmarks. Notably, under WHO criteria, some individuals classified as overweight by Chinese standards were reclassified as normal weight. Further complicating BMI-based assessments, 54 out of 57 students with normal BMI by WHO standards exhibited normal weight obesity (NWO) – a condition where individuals have excessive body fat despite falling within the ‘healthy’ BMI range. This discrepancy reinforces concerns about BMI’s reliability in accurately identifying obesity-related health risks, particularly for individuals near threshold boundaries. Previous research supports this limitation, demonstrating that while a BMI ≥ 30 kg/m^2^ has high specificity (95%−99%) for obesity based on body fat percentage (BF%), its sensitivity drops below 50% for individuals with BMIs between 18.5 and 29.9 kg/m^2^ [22].

Besides, conventional BMI standards may not adequately reflect the unique environmental factors in high-altitude regions such as Tibet. Chronic hypoxia and associated adaptations in body composition adaptations (e.g., altered fat distribution and reduced muscle mass) can significantly modify the relationship between BMI, body fat percentage, and metabolic health risks. For instance, high-altitude exposure may influence dietary behavior, often leading to reduced fat intake and increased consumption of high-calorie, carbohydrate-rich foods [23], which could affect fat accumulation patterns. Although chronic hypoxia is generally linked to impaired protein metabolism and muscle loss, evidence suggests that acute hypoxia, particularly when combined with exercise, may stimulate muscle preservation or growth [24]. This effect is thought to be medicated by the accumulation of metabolic byproducts (e.g., lactate), which lowers pH and may enhance the secretion of growth hormone (GH) [25], thereby promoting muscle adaptation.

The combined impact of muscle catabolism and fat redistribution underscores the importance of evaluating body composition beyond BMI, when assessing obesity and associated health risks. Additionally, high-altitude environments can alter fluid distribution, further diminishing the accuracy of BMI in assessing body fat percentage and increasing the likelihood of misclassifying individuals with NWO. These factors raise important concerns about the validity of conventional BMI thresholds in extreme geographical environments, underscoring the need for altitude-specific health assessment criteria.

Regression analysis in this study revealed distinct gender-specific patterns in the relationship between BMI and body BF%. Females exhibited a consistent positive linear relationship between BMI and BF%. In contrast, a nonlinear association was observed among males characterized by an initially gradual increase in BF% that accelerated markedly beyond a BMI threshold of 19.6. These findings underscore significant gender-based differences in adiposity patterns among the study population.

Notably, the incidence of NWO in this cohort of young adults was 22.2%, with females exhibiting a higher prevalence than males. This finding is consistent with a previous cross-sectional study conducted among Chinese college students in a lowland region [26]. A study by Peng Yue et al. involving 318 plateau-dwelling college students further supported this gender-specific patterns, revealing higher NWO susceptibility among females [27]. Beyond genetic predisposition and age-related influences, behavioral and environmental determinants—including dietary habits, lifestyle behaviors, and psychological factors-also play a significant role in the development of NWO. Supporting this, Cota et al. [28] demonstrated in a study of 506 Brazilian youth (aged 10–19) that females exhibited lower body satisfaction, reduced physical activity levels, and higher sugar consumption compared to males- factors directly linked to NWO. Another study found that females consistently exhibited significantly lower physical activity levels than males, regardless of altitude (i.e., among both lowlanders and highlanders). Notably, this reduced physical activity was accompanied by higher rates of overweight and obesity among females compared to male counterparts [29].

The current results indicated a higher incidence of NWO among migrant adolescents compared to their native counterparts, with migrant females showing a higher BF%. This phenomenon may be attributed to varying durations of high-altitude exposure. Research indicates that prolonged exposure to high-altitude environments leads to reductions in both lean mass and adipose tissue [30], with physiological adaptation typically requiring 7−15 months [31]. We cannot exclude the potential influence of ethnic genetic differences on body composition, given that the native population is Tibetan, whereas most migrants are Han Chinese. Notably, a study comparing genetically matched populations from different altitudes reported divergent findings. Specifically, the study compared individuals from a low-altitude region (230 m in the Amazon basin, n = 230) with those from a high-altitude region (3800 m in the Andes, n = 95). The results showed that high-altitude females had reduced SMM and increased adiposity, with a greater BF% compared to their low-altitude counterparts [32]. Interestingly, our results revealed no significant differences in FFM and SMM between native and migrant young adults, regardless of gender. This finding contrasts with earlier studies that have documented high-altitude-induced alterations in body composition. a meta-analysis [33] revealed significant changes in body composition metrics-particularly lean body mass-at altitudes exceeding 3500m. Short-term hypoxic exposure studies, involving 14 males and 12 females over 21 days, have similarly reported decreases in muscle content [34]. However, the absence of significant differences in our study suggests that such physiological changes may not manifest uniformly in young adults with NWO. These seemingly inconsistent findings may stem from underlying molecular mechanisms. A recent mouse study suggests that chronic hypoxia stabilizes HIF-2α, a transcription factor that impairs muscle regeneration by inhibiting the activation of satellite cells. This inhibition disrupts skeletal muscle repair and may contribute to long-term muscle mass reduction [35]. Thus, the HIF-2α pathway may help explain the observed decrease in SMM among individuals with NWO living at high altitude, especially in populations with limited acclimatization. These findings highlight the complexity of high-altitude acclimatization and suggest that factors such as age, duration of exposure, and pre-existing body composition may influence individual susceptibility to altitude-related changes in lean mass.

The lower BF% observed in native females aligns with existing literature indicating that hypoxic exposure is associated with reduced adiposity. At high altitudes, elevated leptin levels suppress appetite and increase basal metabolic rate, thereby contributing to a sustained negative energy balance and ultimately leading to reduced fat mass [36]. The slightly higher waist-to-hip ratio (WHR) observed in native males compared to migrant males is somewhat intriguing, as previous research has reported no significant differences in WHR between age-matched native and migrant individuals of either gender [37]. However, given the limited sample size of native males with NWO in our study, we cannot exclude the possibility that sampling variability influenced this result.

## Limitations and future directions

This study utilized a relatively large sample size, covering both genders and native/migrant groups to improve the generalizability of the results; however, several limitations should be acknowledged. First, the cross-sectional design precludes causal inferences regarding the relationship between high-altitude hypoxia and normal weight obesity, as it only provides a temporal snapshot of health status. Second, while our sampling strategy enhanced internal validity, the single-region recruitment may limit generalizability to all plateau-dwelling young adults. Furthermore, the absence of lifestyle data (e.g., dietary patterns, physical activity levels) represents a notable constraint, as these variables may substantially influence NWO development. Future research should employ longitudinal designs to examine the temporal dynamics of hypoxia-induced body composition changes, or consider controlled intervention studies to elucidate underlying mechanisms.

## Conclusion

Based on our results, the incidence of NWO among young adults living in a high-altitude region was 22.2%, with a significantly higher prevalence in females compared to males. Additionally, migrant college students exhibited a higher incidence of NWO than their native counterparts. Although the underlying mechanisms are not fully understood, existing research suggests that the hypoxic environments may contribute to an overall reduction in body mass, including fat mass, through mechanisms such as altered appetite regulation, dietary changes, and metabolic adaptations. Additionally, gender-specific physiological responses may influence how body composition adjusts to high-altitude conditions. These findings highlight the importance of incorporating comprehensive body composition assessments into health evaluations in high-altitude regions, as reliance on traditional measures, such as BMI alone, may lead to misdiagnosis in obesity screening.
